# Unilateral secondary middle turbinate and ipsilateral bifid inferior turbinate with presence of uncinate process: Is it true bifid inferior turbinate?

**DOI:** 10.1016/j.radcr.2024.08.087

**Published:** 2024-09-10

**Authors:** Melissa L. Thenata, Anggraini D. Sensusiati

**Affiliations:** Radiology Department, Faculty of Medicine Universitas Airlangga, Jalan Mayjen. Prof. Dr. Moestopo No. 47, Surabaya, Surabaya 60132, Indonesia

**Keywords:** Secondary middle turbinate, Bifid inferior turbinate, Uncinate process, Anatomical variations, Nasal turbinate

## Abstract

Anatomical variations of the nasal turbinate, such as secondary middle turbinate (SMT) and bifid inferior turbinate (BIT), are sporadic. In most cases, SMT and BIT are generally bilateral. Moreover, the uncinate process is unusual in BIT because it is widely acknowledged that BIT could be an abnormality of the uncinate process. However, we found an unusual case of a 19-year-old female with 2 compartments on the right nostril since birth. CT scan and nasal endoscopy revealed unilateral SMT and ipsilateral BIT with the presence of an uncinate process. Therefore, considering the different origins of the uncinate process and inferior turbinate, BIT with the uncinate process can be referred to as true BIT, while BIT without the uncinate process can be referred to as false, double, or accessory inferior turbinate.

## Introduction

The nasal turbinates serve as essential structural elements of the nasal cavity that arise from the lateral nasal wall and consist of soft tissue and bone [1]. It is divided into superior, middle, inferior, and supreme turbinate. The ethmoid bone contains the supreme, superior, and middle turbinates, whereas the inferior turbinates belong to separate pieces of bone [2,3]. The development of the nasal turbinates is a complicated process that occurs during embryologic life, and during this time, numerous anatomical variations are possible to develop [1,4]. The understanding of nasal turbinate variations is essential for patient management as well as assisting surgeons in performing functional endoscopic sinus surgery safely [1,5]. Computed tomography (CT) scan is the first choice in detecting anatomical variations in the sinonasal region. It provides excellent and detailed images and is widely available [4].

The middle turbinate is a significant anatomical structure that serves as an important landmark during endoscopic sinus surgery [6]. Concha bullosa is the most common anatomical variation in the middle turbinate, followed by paradoxical middle turbinate, while secondary middle turbinate (SMT) and accessory middle turbinate are uncommon [4,7]. SMT is a bony protrusion covered by soft tissue originating from the lateral nasal wall, located on the lateral side of the middle turbinate. This term was first described by Khanobthamchai et al. in 1991. The incidence of SMT varies from 0.8% to 6.8% and is usually bilateral [5,7].

In contrast to the middle turbinate, the anatomical variation of the inferior turbinate is often overlooked because endoscopic sinus surgery generally focuses on the middle and superior turbinate. A study conducted by Demir et al. on 376 patients found that 4.5% of patients had inferior turbinate variation, with the most common variation being serrated turbinate, followed by bullous, paradoxical, bifid, and hypoplasia. Bifid inferior turbinate (BIT) was first described by Aksungur et al. in 1999, and based on data compiled by Demir et al. from various studies, the incidence of BIT ranges from 0.03% to 0.91% [8]. There is almost no uncinate process on the BIT in the majority of documented examples [9,10]. In this article, we present an interesting case with SMT and BIT that differs from the majority of cases, unilateral SMT and ipsilateral BIT, with the presence of an uncinate process.

### Case presentation

**Fig. 1:**
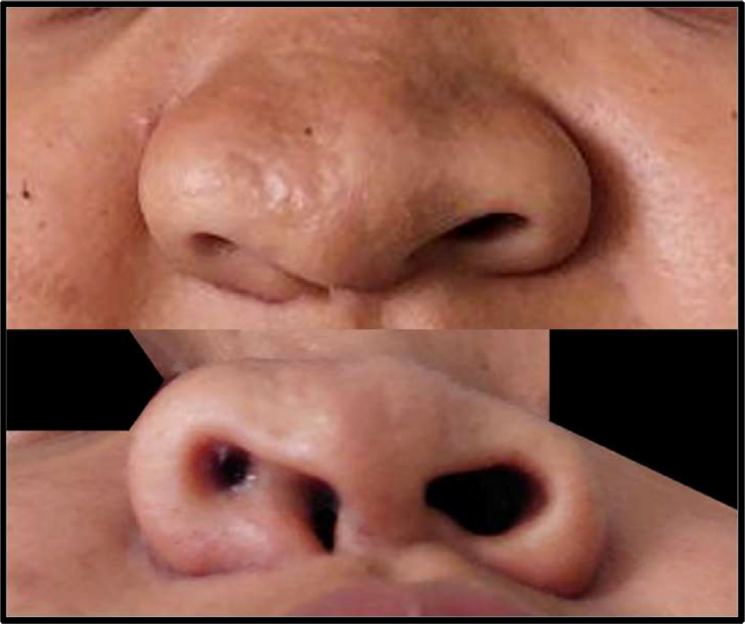
The nose was asymmetrical with the more prominent right side. A partition on the right nostril could be seen dividing the right nostril into 2 compartments.

A 19-year-old female came to the ENT department with 2 compartments on the right nostril since birth that often produce mucus and nasal obstruction. She denied any history of trauma and had never undergone any surgery in the nasal region. During the physical examination, there was a partition in the right nostril (Fig. 1).

**Fig. 2:**
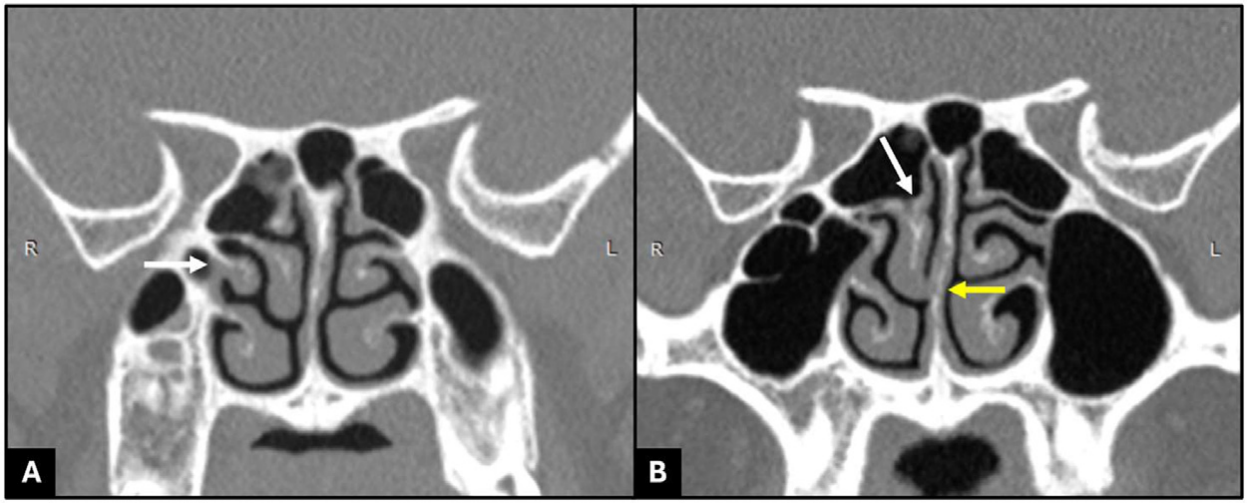
(A) A coronal CT scan of the paranasal sinus showed a right secondary middle turbinate (arrow). (B) The inferior side of the true (medial part) right middle turbinate attaches to the one-third inferior of the nasal septum (yellow arrow), and the superior side merges with the right superior turbinate (white arrow).

The paranasal sinus CT scan showed a right bone structure integrated with the lateral wall of the middle meatus and covered by soft tissue density, which was in line with the image of SMT. The inferior side of the true (medial part) right middle turbinate was attached to the one-third inferior of the nasal septum, and the superior side merged with the right superior turbinate (Fig. 2).

**Fig. 3:**
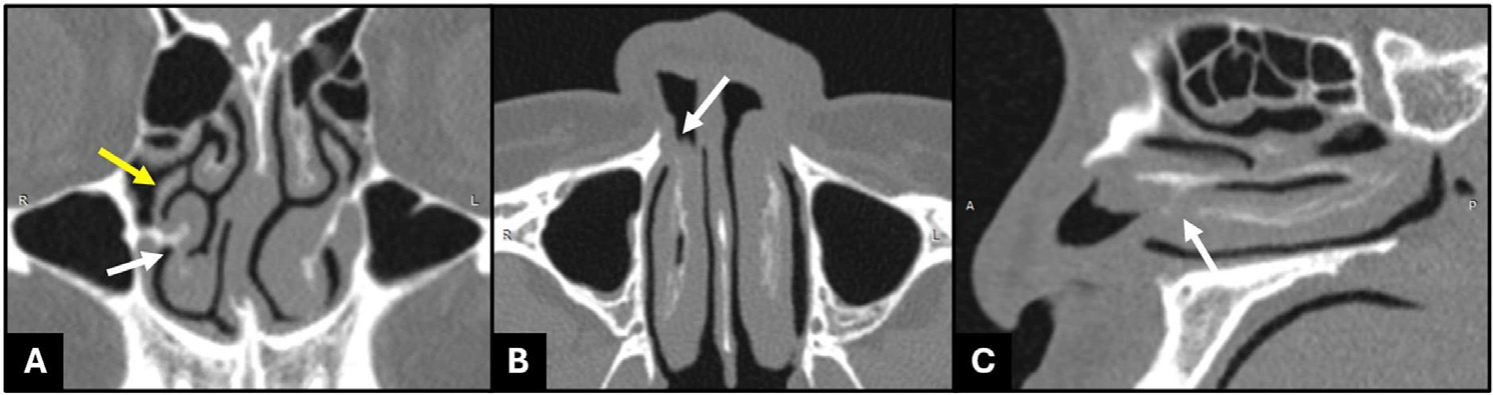
Coronal (A), axial (B), and sagittal (C) paranasal sinus CT depicted 2 inferior turbinates at the right nasal cavity that have the same root, indicating a bifid inferior turbinate (white arrow). The ipsilateral uncinate process (yellow arrow) is present.

There were also 2 inferior turbinates at the right nasal cavity having the same root, indicating a BIT in which the ipsilateral uncinate process was present (Fig. 3).

**Fig. 4:**
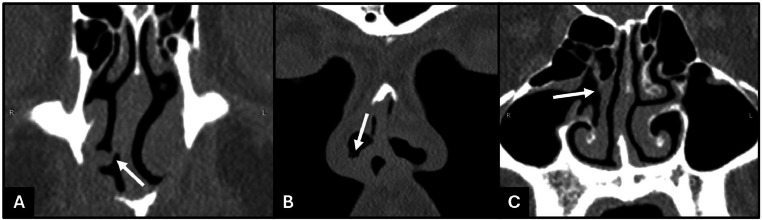
Coronal CT scans showed (A) the inferior part of the bifid inferior turbinate attached to the inferior nasal septum anteriorly, (B) forming a partition that divided the right nostril into 2 compartments, (C) and continued posteriorly to the mid-nasal septum and then attached to the inferior side of the right ethmoid sinus.

The inferior part of BIT has attached to the inferior nasal septum anteriorly, continued posteriorly to the mid-nasal septum, and then attached superiorly to the inferior side of the right ethmoid sinus. The inferior attachment to the inferior nasal septum anteriorly formed a partition that divided the right nostril into 2 compartments (Fig. 4), as seen on physical examination.

**Fig. 5:**
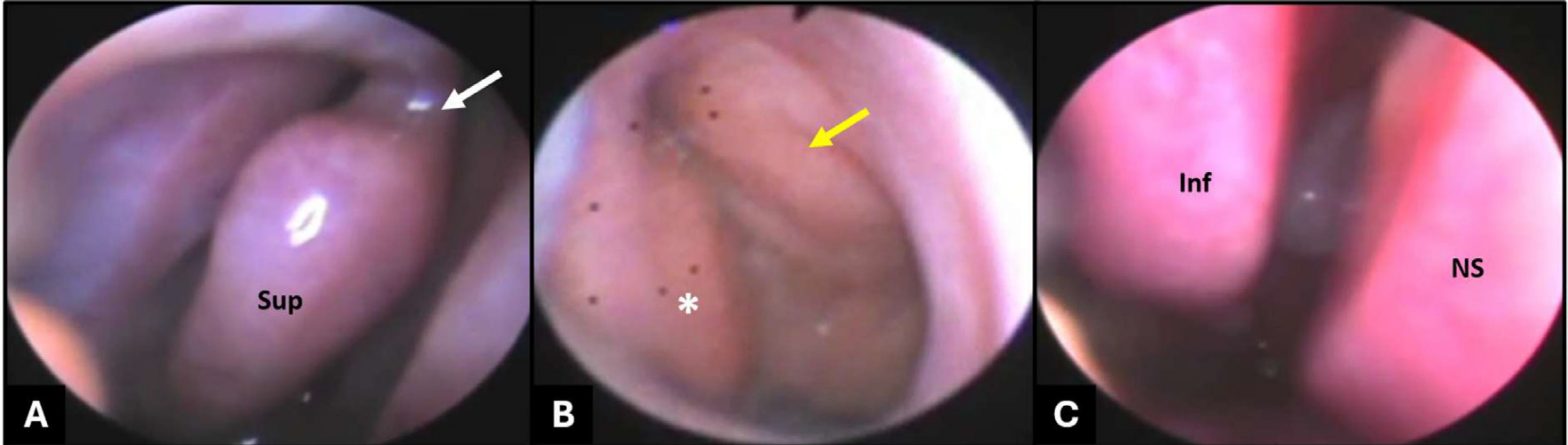
(A, B) Nasal endoscopy from the lateral compartment of the right nostril showed (A) the superior part of the right bifid inferior turbinate and the bifurcation of the right inferior turbinate (arrow); (B) the secondary middle turbinate (asterisk) and true middle turbinate (yellow arrow). (C) The medial compartment of the right nostril showed the inferior part of the right bifid inferior turbinate. Sup, superior; Inf, inferior; NS, nasal septum.

Nasal endoscopy from the lateral compartment of the right nostril confirmed the superior part of the right BIT, the bifurcation of the right inferior turbinate, and the SMT. The medial compartment examination of the right nostril confirmed the inferior part of the right BIT (Fig. 5). The nasal endoscopy confirmed the findings of the CT scan.

## Discussion

The nasal turbinate, which emerges from the lateral nasal wall, is a significant anatomical feature composed of both soft tissue and bone. The ethmoid bone contains the supreme, superior, and middle turbinates, whereas the inferior turbinates arise from separate pieces of bone. Understanding the embryologic development of the lateral nasal wall assists in recognizing its complicated structure [2,3,11].

Based on embryology, the nasal turbinate arises from ethmoturbinal and maxilloturbinal, which occur in the eighth and tenth weeks of fetal life. The maxilloturbinal forms the inferior nasal turbinate, while the ethmoturbinal forms the uncinate process, middle, superior, and supreme turbinate if present [3,11].

Initially, ethmoturbinals have 5 to 6 ridges that are spaced apart by furrows. Each ridge and furrow may merge or vanish during embryonic development, leaving 3 to 4 ridges remaining. The first ridge of ethmoturbinal undergoes partial regression. The upward part forms the agger nasi, and the downward part forms the uncinate process. The second ridge becomes pneumatized and forms the ethmoid bulla. Furthermore, the initial permanent ethmoturbinal emerges from the third ridge and is called the middle turbinate. Throughout this embryologic process, variation of the lateral nasal wall is possible, although rare [5].

SMT was first mentioned by Khanobthamchai et al. in 1991, with incidences of 0.8% to 6.8%. SMT is a bony protrusion of the middle meatus' lateral nasal wall that lies underneath the basal lamella on the lateral side of a typical middle turbinate, and it is presumed that SMT originates from the incomplete anterior wall of the ethmoid bulla. SMT usually does not affect the ostiomeatal complex and is frequently bilateral [5].

The incredibly uncommon abnormality known as BIT was initially reported by Aksungur et al. in 1999. The defining characteristic of BIT is a single turbinate root with 2 inferior turbinates [11]. There was almost no uncinate process on the BIT side in the majority of documented examples. According to literature compiled by Rusu et al. across 7 papers, there were 2 cases of unilateral and 5 cases of bilateral BITs, none of which have an uncinate process [9]. Therefore, it is generally acknowledged that BIT could be an abnormality of the uncinate process caused by significant medial displacement and inferior rotation of the uncinate process, although it does not seem appropriate considering how the uncinate process from first ethmoturbinal and inferior turbinate from maxilloturbinal develop. Hence, the name accessory inferior turbinate is advised [9,10].

In contrast to most cases reported, our case had unilateral SMT and ipsilateral BIT with the presence of an uncinate process. This is similar to the cases reported by Lee and Koh and Rusu et al., where a BIT as well as an uncinate process were found [9,10]; however, we also identified ipsilateral SMT. Rusu et al. stated that their reported case of BIT was the first case of true BIT with an uncinate process, whereas the reported cases of BIT without an uncinate process can be referred to as false BIT or double, as it is a positional variation of the uncinate process that forms the accessory inferior turbinate. The origin of true BIT is maxilloturbinal, and it is unrelated to other embryonic anatomical differences [9]. Based on this statement, our case can also be considered a true BIT due to the existence of an uncinate process even in the presence of SMT.

## Conclusion

The anatomy of the lateral wall of the nasal cavity, with its wide variety of abnormalities, is complex. Paranasal sinus CT scan and nasal endoscopy are paramount in detecting these anatomical abnormalities. The understanding of anatomical variations is essential for patient management as well as assisting surgeons in performing functional endoscopic sinus surgery safely.

## Patient consent

Written consent has been obtained from the patient as no identifiable patient data were included in this case report.
